# Large-scale production of recombinant miraculin protein in transgenic carrot callus suspension cultures using air-lift bioreactors

**DOI:** 10.1186/s13568-020-01079-3

**Published:** 2020-08-13

**Authors:** Yun-Ji Park, Jong-Eun Han, Hyoshin Lee, Yu-Jin Jung, Hosakatte Niranjana Murthy, So-Young Park

**Affiliations:** 1 Department of Horticulture, Division of Animal, Horticultural and Food Sciences, Chungbuk National University, Cheongju, 28644 Republic of Korea; 2 Department of Forest Genetic Resources, National Institute of Forest Science, 39 Onjeong-ro, Suwon, 16631 Republic of Korea; 3 Department of Horticultural Life Science, Hankyong National University, Anseong, 17579 Republic of Korea; 4 Department of Botany, Karnatak University, Dharwad, 580003 India

**Keywords:** *Agrobacterium*-mediated transformation, Alternative sweetener, Carrot callus cultures, Miraculin, Plant cell culture, Recombinant protein

## Abstract

Miraculin, derived from the miracle fruit (*Synsepalum dulcificum*), is a taste-regulating protein that interacts with human sweet-taste receptors and transforms sourness into sweet taste. Since miracle fruit is cultivated in West Africa, mass production of miraculin is limited by regional and seasonal constraints. Here, we investigated mass production of recombinant miraculin in carrot (*Daucus carota* L.) callus cultures using an air-lift bioreactor. To increase miraculin expression, the oxidative stress-inducible *SWPA2* promoter was used to drive the expression of miraculin gene under various stress treatments. An 8 h treatment of hydrogen peroxide (H_2_O_2_) and salt (NaCl) increased the expression of miraculin gene by fivefold compared with the untreated control. On the other hand, abscisic acid, salicylic acid, and methyl jasmonate treatments showed no significant impact on miraculin gene expression compared with the control. This shows that since H_2_O_2_ and NaCl treatments induce oxidative stress, they activate the *SWPA2* promoter and consequently up-regulate miraculin gene expression. Thus, the results of this study provide a foundation for industrial-scale production of recombinant miraculin protein using transgenic carrot cells as a heterologous host.

## Key points

- Miraculin protein (a taste modifier) gene tagged with *SWAP2* promoter was cloned to carrot cells.
- Transformed carrot cells were cultured in air-lift bioreactors for the production of recombinant miraculin protein.
- Effect of hydrogen peroxide, salt (NaCl), abscisic acid, salicylic acid and methyl jasmonate were tested on miraculin gene expression.
- Treatment of hydrogen peroxide, NaCl, increased the expression of miraculin gene by fivefold compared to control.

## Introduction

Miraculin is a sweet protein produced from the fruit of *Synsepalum dulcificum*. Miraculin protein has attracted much attention as a natural alternative sweetener because it has a taste-modifying activity that transforms sour taste into sweet taste and is low in calories (Jin et al. 2013; Ezura and Hiwasa-Tanase 2018). *S. dulcifica* is native to West Africa and is difficult to grow on a large scale because of climatic limitations, thus making the mass production of miraculin difficult. Therefore, large-scale production of miraculin for industrial purposes requires an investigation of alternative production systems using heterologous hosts (Sun et al. 2007; Yano et al. 2010).

Recently, plant systems have been studied as a platform for the production of recombinant proteins. Plants have low risk for mammalian pathogens and cost-effective production process as heterologous production systems (Huang and McDonald 2012). Methods for producing recombinant proteins in plants include *Agrobacterium*-mediated transformation and transient expression via agroinfiltration, biolistics, and electroporation. In the recent past, *Agrobacterium*-mediated transformation is the most common method of plant transformation (Huang et al. 2018). In the present study, we have used *SWPA2* promoter (obtained from sweet potato) which could over express the tagged gene under stress conditions and it has used successfully to transform the useful genes into several plants (Kim et al. 2003, 2004).

Plant cell culture has recently been considered as an alternative system for the production of recombinant proteins (Hellwig et al. 2004) and has the following advantages: (1) high growth rate, (2) ease of transformation, and (3) high protein production capacity (Corbin et al. 2016). Plant cell culture in bioreactors enables efficient, large-scale, and uniform production of recombinant proteins under controlled conditions (Huang and McDonald 2009). Three different types of bioreactors have been used for recombinant protein production in plant cell suspension cultures including stirred-tank bioreactors, air-lift bioreactors, and membrane bioreactors (Huang and McDonald 2009), and the yield of recombinant protein varies among these bioreactors. Air-lift bioreactor is suitable for growing plant cell cultures that are sensitive to physical stress because it minimizes the physical stress that occurs when cells move through the incubator (Eibl and Eibl 2008). It also has a lower operating cost than other types of bioreactors and has the advantage of easy scale-up for large-scale plant cell culture (Huang and McDonald 2012). Because of these advantages, López et al. (2014) and Corbin et al. (2016) used bioreactor cultures for recombinant protein production. However, there have been no reports on the use of bioreactors for the production of sweet proteins, such as miraculin. Therefore, research is needed to optimize bioreactor culture conditions for the mass production of miraculin in transgenic cell cultures.

In this study, we investigated recombinant miraculin production in carrot (*Daucus carota* L.) callus transformed by *Agrobacterium tumefaciens* and cultured in air-lift bioreactors. This study aims to increase the expression of the miraculin gene, driven by the *SWPA2* promoter, in transgenic carrot callus through abiotic stress and light quality treatments, and to optimize the bioreactor culture system for mass production of miraculin.

## Materials and methods

### Plant material

Carrot callus was infected with *A. tumefaciens* carrying the FLAG-tagged miraculin gene (accession no. D38598) driven with stress-inducible *SWPA2* promoter (Kim et al. 2003). Transgenic callus (TC) lines were selected on callus induction medium [CI medium: Murashige and Skoog (MS) solid medium with 0.25 g L^−1^ casein hydrolysate; 0.22 mg L^−1^ BA; 1 mg L^−1^ 2,4-d; 30 g L^−1^ sucrose; and 2.4 g L^−1^ gelrite] containing 100 µg mL^−1^ kanamycin. TC lines (TC1-TC18) were cultured in CI medium at 24 ± 1 °C in the dark for 2 weeks for further study. After 2 weeks, TC lines (TC3, TC13, TC17, TC18) with high miraculin expression and cell growth were selected and cultured in liquid CI medium for cell suspension culture at a culture density of 40 g L^−1^ at 100 rpm.

### Abiotic stress treatments

To analyze the effect of abiotic stress on miraculin gene expression, TC3 line was treated with five stress factors including hydrogen peroxide (H_2_O_2_) at 220, 440, and 880 µM, salt (NaCl) at 50, 100, and 200 mM, and abscisic acid (ABA), salicylic acid (SA), and methyl jasmonate (MeJA) at 50, 100, 200 µM. To conduct these treatments, TC3 was pre-cultured in liquid CI medium at a culture density of 40 g L^−1^ for 3 days. Abiotic stress treatments were carried out at 24 ± 1 °C at 100 rpm for 8 h in the dark, with three replicates per treatment. Sampling was performed at 0 h (untreated control) and 8 h after treatment, and samples were used for miraculin expression analysis.

### Light quality treatment

To determine the effect of light quality on miraculin gene expression, TC3 line was cultured under six different light qualities: dark (D), white LED (PLLCC 5450 6-pin; Its-well Co., Incheon, Korea) (W), red light (R), blue light (B), red1: blue1 (RB), red1: green1: blue1 (RGB). In each treatment, light intensity was maintained at 65–75 µmol m^−2^ s^−1^, with a 16 h light/8 h dark photoperiod. TC3 was cultured in CI medium at 24 ± 1 °C under various light qualities for 3 weeks. Each light quality treatment was performed in triplicate. Miraculin gene expression was analyzed 1 week after treatment, and the fresh weight, chlorophyll content, and carotenoid content of TC were analyzed after 3 weeks of culture.

### Analysis of chlorophyll and carotenoid contents

Chlorophyll and carotenoid contents of TC3 used to light quality treatment were analyzed as described previously (Boamponsem and Leung 2017). Briefly, 100 mg TC previously frozen in liquid nitrogen was pulverized with Tissue-Lyser II (QIAGEN, Germany) in 1 mL of 80% acetone. After centrifugation at 13,000 rpm for 20 min, the supernatant was transferred to a new tube, and absorbance was measured at 470, 645, and 663 nm using Optizen POP (Mecasys Co., Korea).

### Bioreactor culture of transgenic callus

To test the possibility of mass production of miraculin, TC3 was cultured in a 1-L air-lift bioreactor containing CI medium at a culture density of 40 g L^−1^. Culture was carried out at 24 ± 1 °C in the dark, and aeration volume was adjusted to 0.1 vvm. Sampling was carried out every 6 days, and the experiment was terminated after 18 days. Samples were cryopreserved at − 70 °C, and used for miraculin gene expression and protein production analyses.

### Miraculin gene expression analysis

Reverse transcription quantitative real-time PCR (RT-qPCR) was performed to analyze miraculin expression according to abiotic stress treatment and period of bioreactor culture in transgenic callus. TC3 (100 mg) cryopreserved in liquid nitrogen was used to extract total RNA and pulverized with Tissue-Lyser II (QIAGEN, Germany). Total RNA was extracted from the transgenic callus following the protocol of the NucleoSpin^®^ RNA Plant and Fungi kit (MACHEREY–NAGEL GmbH & Co., Germany). The extracted total RNA was used for cDNA synthesis using ReverTra Ace^®^ qPCR Master Mix (TOYOBO, Japan), which was then diluted to a concentration of 100 ng μL^−1^. For RT-qPCR, we mixed a 20 μL reaction containing 10 μL TB Green Premix Ex *Taq* (Takara, Japan), 0.5 μL of each forward primer and reverse primer, 7 μL sterile distilled water, and 2 μL of cDNA and performed miraculin gene expression analysis using the CFX96 Touch™ Real-Time PCR System (Bio-Rad, California). The miraculin gene was amplified using primers miraculin-RT Fw (5′-CATCAATTTCTCGGCGTTCAT-3′) and miraculin-RT Rv (5′-AAACCACTACCACAAAACTCCT-3′). As a reference, the *DcActin* gene was used for normalization.

### Miraculin protein analysis

SDS-PAGE analysis was performed to confirm the production of miraculin protein in TC cultured in a bioreactor. TC3 (200 mg) was pulverized with Tissue-Lyser II (QIAGEN, Germany) in 400 µL protein extraction buffer (500 μL of 3 M NaCl, 200 μL of 1 M Tris (pH 7.5), 20 μL of 0.5 M EDTA, 1 mL of 10% Triton X-100, 100 μL of 10% SDS, 10 μL of 1 M dithiothreitol (DTT), and 10 μL of protease inhibitor cocktail. After pulverizing, the supernatant separated from the sample was transferred to a new tube by centrifugation at 13,500 rpm. The supernatant was used for SDS-PAGE analysis and protein concentration was examined according to Bradford assay (1976). The TSP was quantitated at the concentration of 30 μg μL^−1^ with distilled water, and then we mixed 24 μL of the diluted TSP and 6 μL of 5X protein sample buffer (ELPIS, Korea). Samples were incubated at 95 °C for 5 min and loaded onto an Express-PlusTM PAGE Gel (GenScript, USA) containing Bis–Tris 10% MOPS buffer. Then, TSP was separated by SDS-PAGE using a Mini-Slab Chamber (ATTO, Japan) at 140 V for 55 min; bright BAND (ZEPTO, Taiwan) was used as a protein marker. After the electrophoresis was completed, the gel was dyed with EzStainAQua (ATTO, Japan) solution for 1 h and washed three times with distilled water for 30 min each.

### Enzyme-linked immunosorbent assay (ELISA)

The yield of FLAG-tagged miraculin protein was quantified by ELISA using the DYKDDDDK-Tag Detection ELISA Kit (Cayman, Germany). Absorbance was measured at 450 nm using Optizen POP (Mecasys Co., Korea).

### Statistical analysis

One-way analysis of variance (ANOVA) analysis was used to indicate significant differences in the data. Statistical processing of the data was done by Duncan’s multiple test at 5% significance level. Analyzes were performed using the SAS program (version 9.4; SAS Institute Inc. Cary, NC, USA).

## Results

**Fig. 1:**
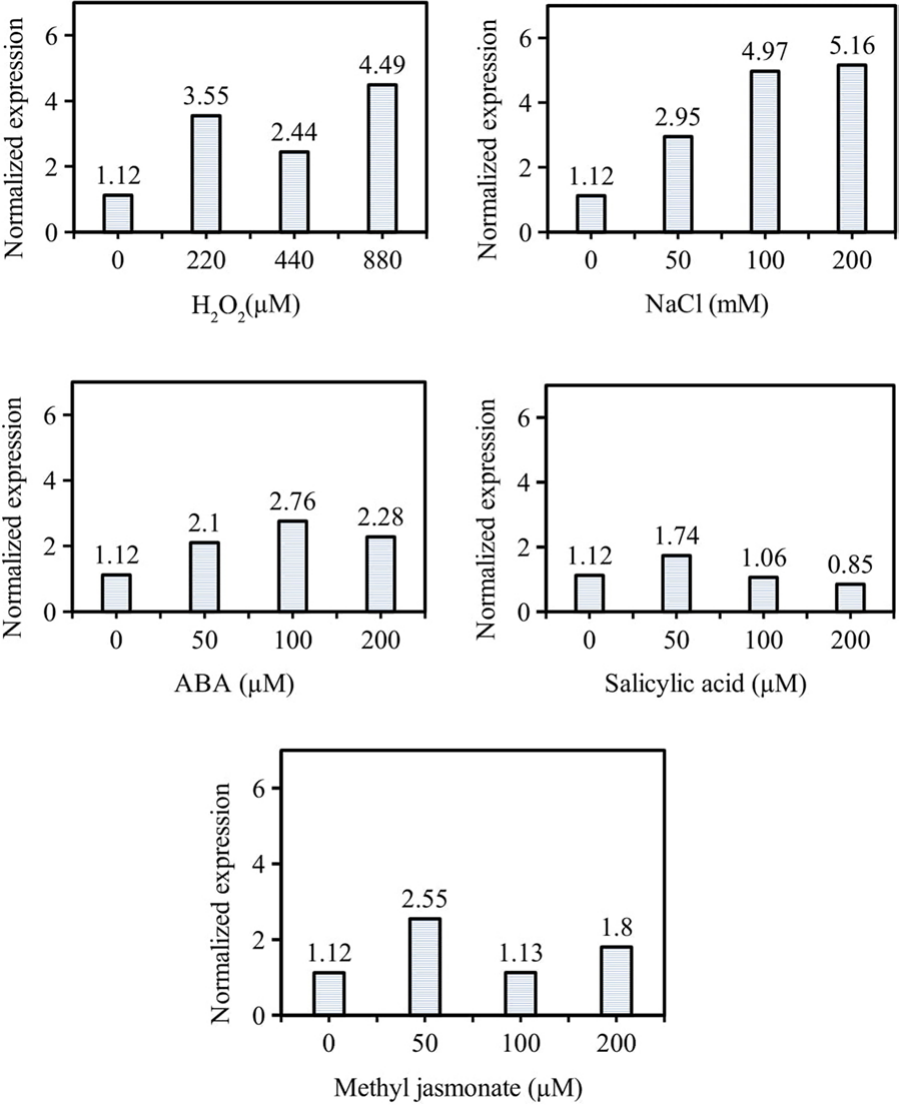
Miraculin gene expression in transgenic callus under abiotic stress treatments. Data represent mean ± SE (*n* = 5). Different lowercase letters indicate statistically significant differences at *P* < 0.05 (Duncan’s multiple range test)

Treatment of TC3 liquid suspension culture with different concentrations of H_2_O_2_ and NaCl for 8 h significantly up-regulated the expression of the miraculin gene compared with the control (Fig. 1). Among various treatments, 880 µM H_2_O_2_ and 200 mM NaCl resulted in the highest miraculin expression, and treatment with 200 mM NaCl showed the highest expression rate (fivefold greater than the control). On the other hand, ABA, SA and MeJA treatments did not show noticeably difference in miraculin expression compared to the control (Fig. 1).

**Fig. 2:**
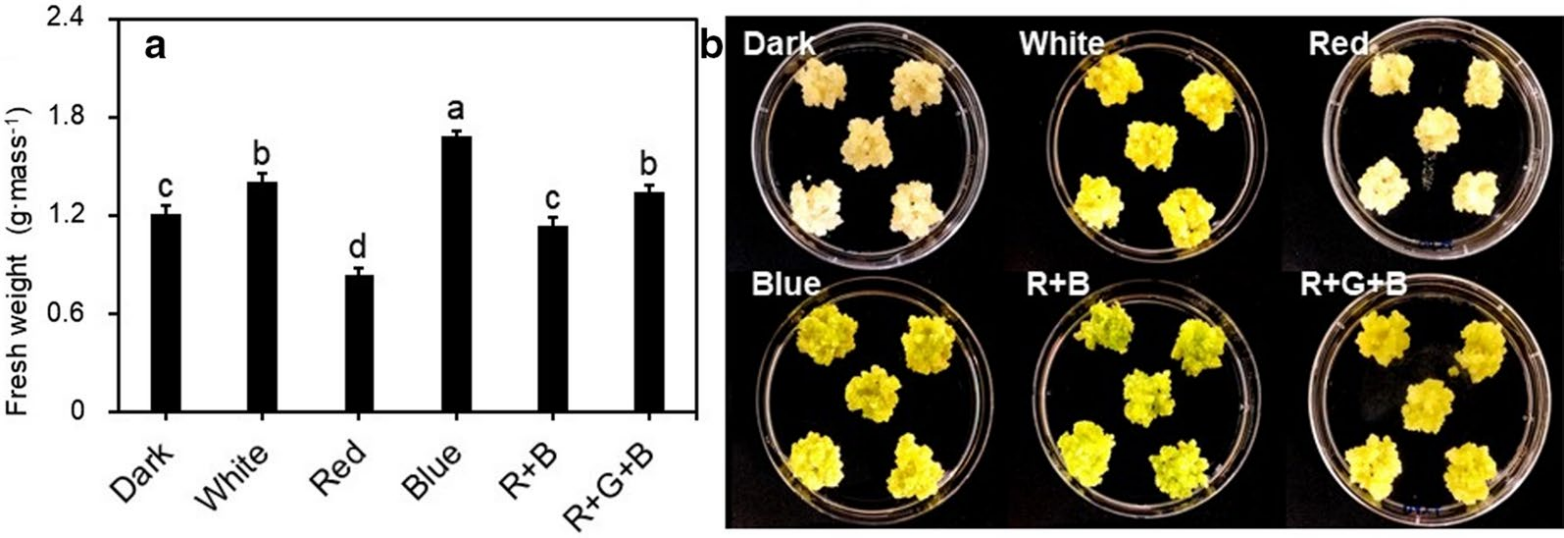
Effect of light quality on the fresh weight of transgenic callus. **a** Fresh weight of callus cultured under different light quality; **b** callus growth after 4 weeks of culture. Data represent mean ± SE (*n* = 5). Different lowercase letters indicate statistically significant differences (*P* < 0.05; Duncan’s multiple range test)

**Fig. 3:**
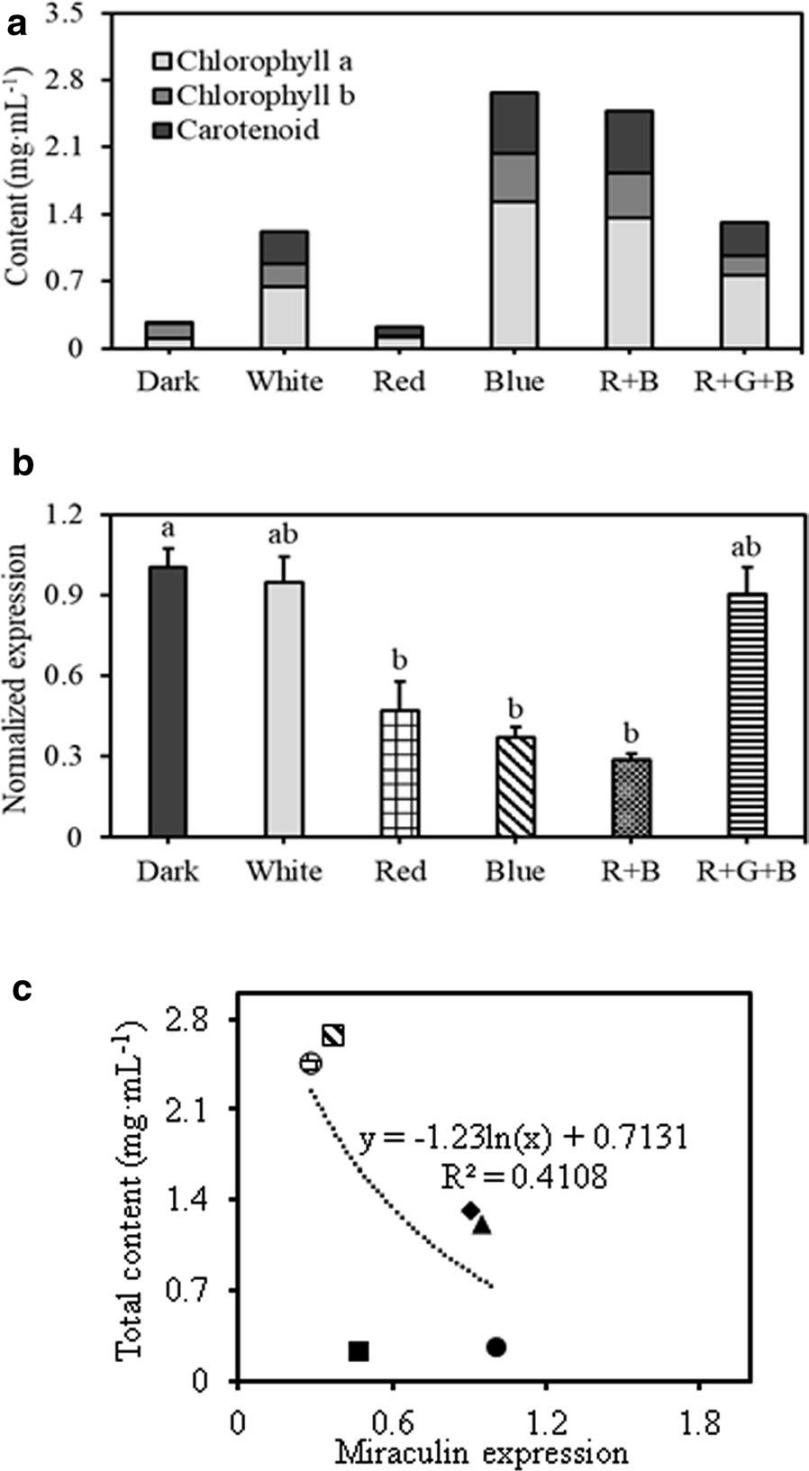
The relation between chlorophyll content and miraculin expression from transgenic carrot callus cultured under different light qualities. **a** Chlorophyll and carotenoid contents of transgenic callus; **b** Miraculin expression in transgenic callus; **c** Correlation analysis of chlorophyll, carotenoid with miraculin expression. Transgenic callus grown in the dark served as a control. Data represent mean ± SE (*n* = 5). The value of control was taken as 1 to compare with transformed callus line. Different lowercase letters indicate statistically significant differences (*P* < 0.05; Duncan’s multiple range test)

The TC3 cells were cultured under six different types of lights to verify the effect of light quality. Fresh weight of TC was the highest (1.7 g mass^−1^) under blue light (B treatment) and lowest (0.8 g mass^−1^) under red light (R treatment), indicating a 2.0-fold difference between these two treatments (Fig. 2). Chlorophyll and carotenoid contents were highest in W, RB, and RGB treatments and highest in B treatment (Fig. 3a). Expression of the miraculin gene was optimum in dark condition and relatively high in W and RGB treatments (Fig. 3b). Miraculin expression was associated with pigment contents. Interestingly, treatments associated with low pigment contents showed high miraculin expression (Fig. 3b). Moreover, correlation analysis confirmed a negative correlation between miraculin expression and chlorophyll content (Fig. 3c).

**Fig. 4:**
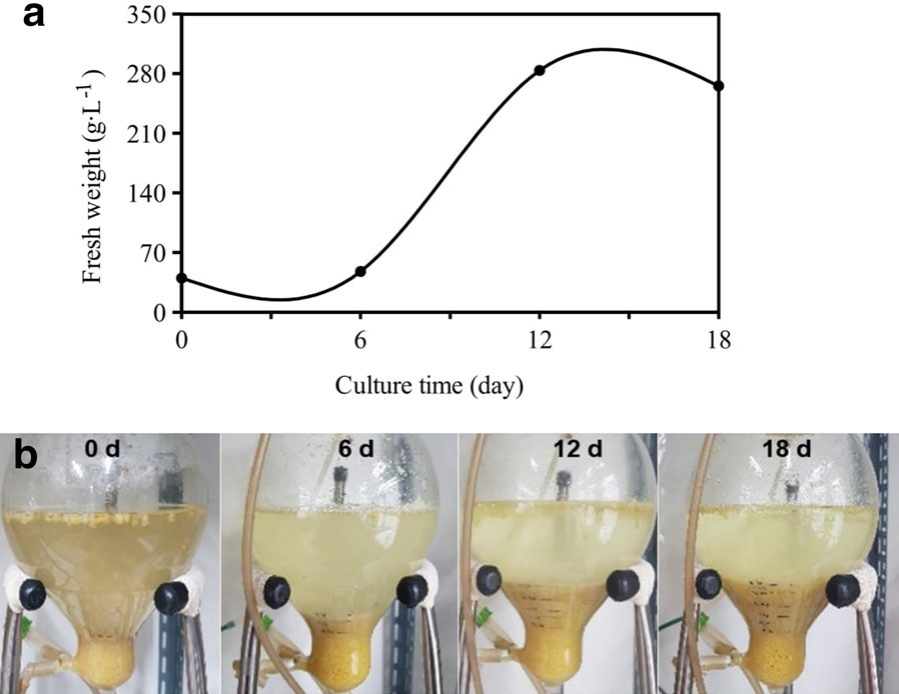
Cell growth of transgenic carrot callus in air-lift bioreactor during culture period. **a** Packaged cell growth in bioreactor; **b** Fresh weight per liter medium

**Fig. 5:**
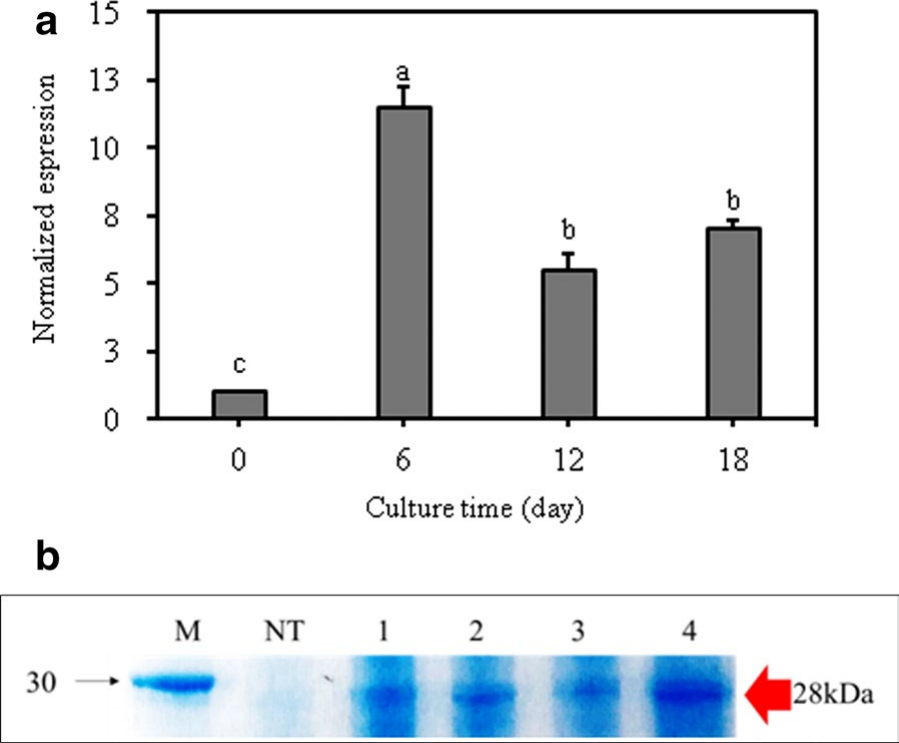
Miraculin expression and protein analysis by SDS-PAGE of transgenic callus harvested from bioreactor culture during culture period. **a** miraculin gene expression; **b** SDS-PAGE analysis of miraculin protein. In A, the 0-day time point served as a control. Data represent mean ± SE (*n* = 5), and different lowercase letters indicate statistically significant differences (*P* < 0.05; Duncan’s multiple range test). The value of control was taken as 1 to compare with transformed callus line

TC3 cell lines were cultured in airlift bioreactors and analyzed the fresh weight and miraculin expression. The fresh weight of TC3 increased slowly until day 6 since the start of culture (lag phase), increased rapidly from day 6 to day 12 (exponential phase), and then decreased thereafter (Fig. 4a, b). Miraculin expression was also the highest at 6 days of culture and decreased at 12 days of culture (Fig. 5a).

Analysis of the TSP by SDS-PAGE confirmed the production of miraculin protein according to the bioreactor culture period. The results of SDS-PAGE showed expression of miraculin protein in all the cell lines (Fig. 5b). We also performed ELISA to confirm the yield of miraculin protein in the bioreactor cultures. The content of miraculin was 0.024 µg µL^−1^ (0.98% of TSP) at 6 days and 0.013 µg µL^−1^ (0.64% of TSP) at 12 days (Table 1). These results indicate that the production of miraculin in bioreactors increased approximately 30- to 90-fold compared with the recombinant miraculin content produced at 0-day of TC (0.01% of TSP) (Table 1).

**Table 1:** Concentration of miraculin protein extracted from transgenic callus cultured in an air-lift bioreactor

Culture period (day)	Total soluble protein (TSP) (µg/µL)	Purified miraculin protein (µg/mL)	Purity (% TSP)
0	2.366	0.0001	0.01
6	2.412	0.0237	0.98
12	2.070	0.0132	0.64
18	1.805	0.0013	0.07

## Discussion

In the present study, miraulin gene which was tagged to *SWPA2* promoter, cloned carrot cell lines and transformed cell lines were used for production of recombinant protein. The effect of H_2_O_2_, NaCl, ABA, SA and MeJA were tested on the expression of miraculin gene in the transformed (TC3) cell line (Fig. 1). Among various treatments, 880 µM H_2_O_2_ and 200 mM NaCl resulted in the highest miraculin expression, and treatment with 200 mM NaCl showed the highest expression rate (fivefold greater than the control). However, the ABA, SA and MeJA treatments did not show considerable difference in miraculin expression compared to the control (Fig. 1). Kim et al. (2004) reported that the stress-inducible *SWPA2* promoter used in this study is regulated by peroxidase (POD), which is induced by oxidative stress. Additionally, H_2_O_2_, one of the reactive oxygen species (ROS), is not only a reactive substance for POD but also a signaling molecule directly involved in various stresses such as oxidative stress (Neill et al. 2002). Stress caused by higher concentrations of NaCl is known to induce the production of ROS in cells (Tian et al. 2016), which causes oxidative stress, leading to an increase in the content of POD to reduce ROS from plants (Foyer and Noctor 2005). These data suggest that the treatment of TC lines with NaCl and H_2_O_2_, which directly or indirectly induce oxidative stress, increases the activity of the *SWPA2* promoter, resulting in high miraculin expression.

In another set of experiments, we verified the effect of various light sources on TC3 cell line for the production of miraculin protein. In general, dark condition was maintained during the cell cultures. However, in current experiments, B, RGB, and W treatments resulted in a highest fresh weight cells. B treatment is closely related to microtubule biosynthesis genes, serine carboxypeptidase, chlorophyll biosynthesis, sugar degradation, and expression of various resistance-related genes (Li et al. 2017; Batista et al. 2018). Li et al. (2017) demonstrated that B treatment increases protoplast development and leaf growth in grape. The increase in the fresh weight of transgenic carrot cell line (TC3) upon treatment with blue light might be due to increased biosynthesis of cell growth-related substances such as intracellular microtubules. Plant cells possess photoreceptors such as phytochrome and cryptochrome, and the amount of light absorption by these photoreceptors varies with the light environment (Jiao et al. 2007). Cryptochrome recognizes the wavelength range from ultraviolet A (UV-A) to the blue light region, and phototropin, a photoreceptor, especially absorbs light in this region. Blue light absorbed by phototropin promotes chlorophyll biosynthesis and chloroplast development, resulting in the highest chlorophyll and carotenoid contents (Batista et al. 2018).

The performance of transformed cell line TC3 in bioreactor cultures was studied and results revealed that the fresh weight of TC3 increased slowly until day 6 since the start of culture (lag phase), increased rapidly from day 6 to day 12 (exponential phase), and then decreased thereafter (Fig. 4a, b). Miraculin expression was also the highest at 6 days of culture and decreased at 12 days of culture (Fig. 5a). Polymenis and Aramayo (2015) found that protein is one of the most abundant macromolecules in actively dividing cells. Therefore, it was thought that the expression of miraculin was the highest at 6 days after bioreactor culture in which cell division became active. Furthermore, TC3 cell lines were cultured in airlift bioreactors showed better biomass and miraculin expression (Fig. 5a and Table 1). According to Egelkrout et al. (2012), the efficiency of foreign protein production varies with the host plant, transformation method, and protein storage location. The host plant is one of the most important factors because the amount of foreign protein production depends on the content of endogenous proteins. Because the total amount of protein that plants can biosynthesize is limited, the higher the endogenous protein content, the higher the foreign protein content. When the total protein content is limited, the production of foreign protein often competes with that of endogenous proteins (Egelkrout et al. 2012). Thus, the negative correlation detected in this study between miraculin expression and chlorophyll content probably resulted from the primary metabolite competition between endogenous chlorophyll biosynthesis and recombinant miraculin expression. Previous studies on recombinant protein production have not reported correlations between expression of recombinant protein and biosynthesis of chlorophyll and carotenoids. Thus, this is the first report on the correlation between recombinant protein production and pigment biosynthesis. Sun et al. (2006, 2007) developed transgenic lettuce and tomato plants for the production of miraculin protein. However, the level of miraculin accumulation was varied in transgenic progenies (To, T1, T2 and subsequent progenies). Further, Sun et al. (2006, 2007) reported the variability in miraculin concentrations in leaves/fruits of transgenic lettuce and tomato plants; this hampers the constant and continuous production of miraculin protein. In the current studies, we have demonstrated the cloning of miraculin gene to carrot callus lines and these lines could be used for constant and continuous production of miraculin protein within short period of time.

In conclusion, in the present study, we attempted to increase miraculin expression in transformed carrot cell lines using various abiotic stress treatments. Miraculin expression was up to fivefold higher in TC treated with H_2_O_2_ and NaCl compared with control. Additionally, high miraculin expression was observed when cultured under W, RGB, and dark conditions. Moreover, air-lift bioreactor increased the production of miraculin protein by 30- to 90-fold compared with semi-solid culture, confirming the possibility of mass production. Overall, this study provides basic data for the industrial use of miraculin using transformed carrot cell lines.

## Data Availability

All the relevant data is presented in the manuscript.
